# Patterns and stability of long-term adherence in continuous positive airway pressure therapy for obstructive sleep apnea: a cohort study

**DOI:** 10.1007/s11325-025-03418-9

**Published:** 2025-07-15

**Authors:** Karin Jeppesen, Asbjørn Kørvel-Hanquist, Donna Lykke Wolff, Sofie Ronja Petersen, Preben Homøe, Eva Kirkegaard Kiær, Poul Jørgen Jennum, Helene Skjøt-Arkil

**Affiliations:** 1 Department of Ear, Nose and Throat Surgery, University Hospital of Southern Denmark, Sønderborg, Denmark; 2 Department of Regional Health Research, University of Southern Denmark, Odense, Denmark; 3 Department of Otorhinolaryngology and Maxillofacial Surgery, Zealand University Hospital, Køge, Denmark; 4 Department of Clinical Medicine, Faculty of Health Sciences, University of Copenhagen, Copenhagen, Denmark; 5 Department of Clinical Research, University Hospital of Southern Denmark, Aabenraa, Denmark; 6 Department of Otorhinolaryngology, Head and Neck Surgery, and Audiology, Copenhagen University Hospital, Copenhagen, Denmark; 7 Danish Center for Sleep Medicine, Department of Clinical Neurophysiology, Copenhagen University Hospital, Copenhagen, Denmark

**Keywords:** Obstructive sleep apnea (OSA), Continuous positive airway pressure (CPAP), Virtual data, Adherence, Sleep clinic

## Abstract

**Purpose:**

• Despite being the gold standard for treating obstructive sleep apnea (OSA), continuous positive airway pressure (CPAP) therapy faces a persistent challenge: patient adherence. This study aimed to investigate long-term adherence and stabilisation patterns after initiating CPAP therapy.

**Methods:**

• We conducted a multicenter cohort study using tele-monitored CPAP data from adult patients treated for at least 24 months at two Danish sleep clinics. These data were combined with local hospital administrative data and national registry data.

**Results:**

• The study included data from 1,907 OSA patients who had initiated CPAP. The median age was 55 years (SD 12.5), and 26% were females. After long-term CPAP therapy, 45% of the OSA patients had achieved high adherence to CPAP, while 39% were CPAP non-adherent, and 16% of the patients were in the low-adherent group. The probability of high adherence after two years was 79% if the patient achieved high adherence within the first month. All groups showed stability within the first three months after CPAP initiation, particularly for the CPAP non-adherent patients and the patients with high adherence.

**Conclusion:**

• We found that less than half of the OSA patients achieved high adherence to CPAP therapy 24 months after CPAP initiation. Patients who achieved high adherence within the first month were more likely to maintain it.

**Supplementary Information:**

The online version contains supplementary material available at 10.1007/s11325-025-03418-9.

## Background

Obstructive sleep apnea (OSA) is an increasingly common condition, affecting approximately one billion patients worldwide. The rise is thought to be due to the worldwide obesity epidemic and the ageing population [1, 2]. Untreated OSA is associated with an increased risk of morbidity, such as diabetes, cardiovascular diseases, stroke and a higher risk of mortality, traffic accidents, decreased quality of life, and significant socioeconomic consequences [3–6]. Continuous positive airway pressure (CPAP) therapy is the standard first-line treatment for OSA patients. However, only approximately 40–65% of the patients succeed with the treatment [7–9], which is critical for efficacy since the duration of CPAP use is positively correlated with improved outcomes [10]. Reasons for non-adherence to CPAP therapy are multifactorial and include physiological issues such as nasal congestion, mask leak, and discomfort from pressure settings, as well as psychological challenges such as insomnia, claustrophobia, and the additional burden of coping with a chronic condition [11].

Telemonitoring of CPAP therapy provides access to adherence data from the first day of therapy. In recent years, a few but large studies have analysed this type of data [12–14]. However, the data are often anonymous and only evaluated up to one year after CPAP initiation. Therefore, studies on long-term CPAP usage patterns based on detailed telemonitored adherence data are requested.

While CPAP therapy remains the cornerstone of OSA management, the current trend favors personalised treatment plans designed to address patient-specific needs and boost adherence. Consequently, alternative treatment modalities are now being considered earlier after the OSA diagnosis [15]. Determining when a patient is considered stable regarding a high, low, or non-adherence pattern involves investigating the timeline and factors influencing adherence patterns. This information could help in developing an algorithm for personalised treatment of patients non-adherent to CPAP therapy.

### Aim and objectives

This study aims to investigate long-term adherence and stabilisation patterns after initiating CPAP therapy. The research questions are:

- What is the proportion of CPAP-treated patients with high adherence, low adherence, or no adherence during long-term treatment?
- What is the transition probability between the levels of adherence during the first year of therapy compared to long-term?
- When, if observed, do adherence levels achieve stability?

## Methods

### Study design

We conducted a multicenter cohort study using two years of retrospective data collected from OSA patients with telemonitored CPAP combined with local hospital and national registry data. The study was reported in accordance with the STROBE (Strengthening the Reporting of Observational studies in Epidemiology) guidelines [16].

### Setting and data sources

This study included OSA patients from the Sleep Clinic at the Zealand University Hospital in Køge and Hospital Sønderjylland in Sønderborg. The study period started the day the Sleep Clinics began telemonitored CPAP therapy (1 January 2015 and 1 January 2020, respectively) and went until 31 December 2022.

Patients were offered CPAP therapy as the first-line treatment if they were diagnosed with OSA after a medical examination conducted either by a primary care ear, nose and throat (ENT) doctor or by an ENT doctor at one of the sleep clinics, combined with cardiorespiratory monitoring or partial polygraphy confirming the diagnosis. All patients were offered CPAP with telemonitoring, which was used solely to perform remote technical adjustments when patients contacted the sleep clinics. There was no proactive monitoring or outreach based on adherence data. If CPAP therapy was not successful for the patient, other possibilities, such as a mandibular advancement device (MAD), surgery or a positional trainer, were discussed. In Denmark, healthcare services are free and tax-financed; however, MAD and positional trainers are usually self-funded by patients and rarely covered by public healthcare. During the study period, OSA-related surgery was rarely performed; therefore, CPAP was the only treatment option for the majority of patients.

Data on adherence were collected from the CPAP machines as part of the medical record from ResMed AirView^®^ using serial numbers. This information was combined with national data on hospital affiliation, hospital contacts, and medical procedures collected from the Danish National Patient Registry (DNPR) and linked using unique personal identification numbers [17].

### Population

All OSA-diagnosed adults (≥ 18 years) with prescribed CPAP machines connected to telemonitoring technology were considered eligible. Patients were excluded if they (a) did not have two full years of telemonitoring or (b) were current CPAP users who had previously had a CPAP machine without telemonitoring and were now treated with a new machine with telemonitoring.

### Variables and definitions

**Fig. 1:**
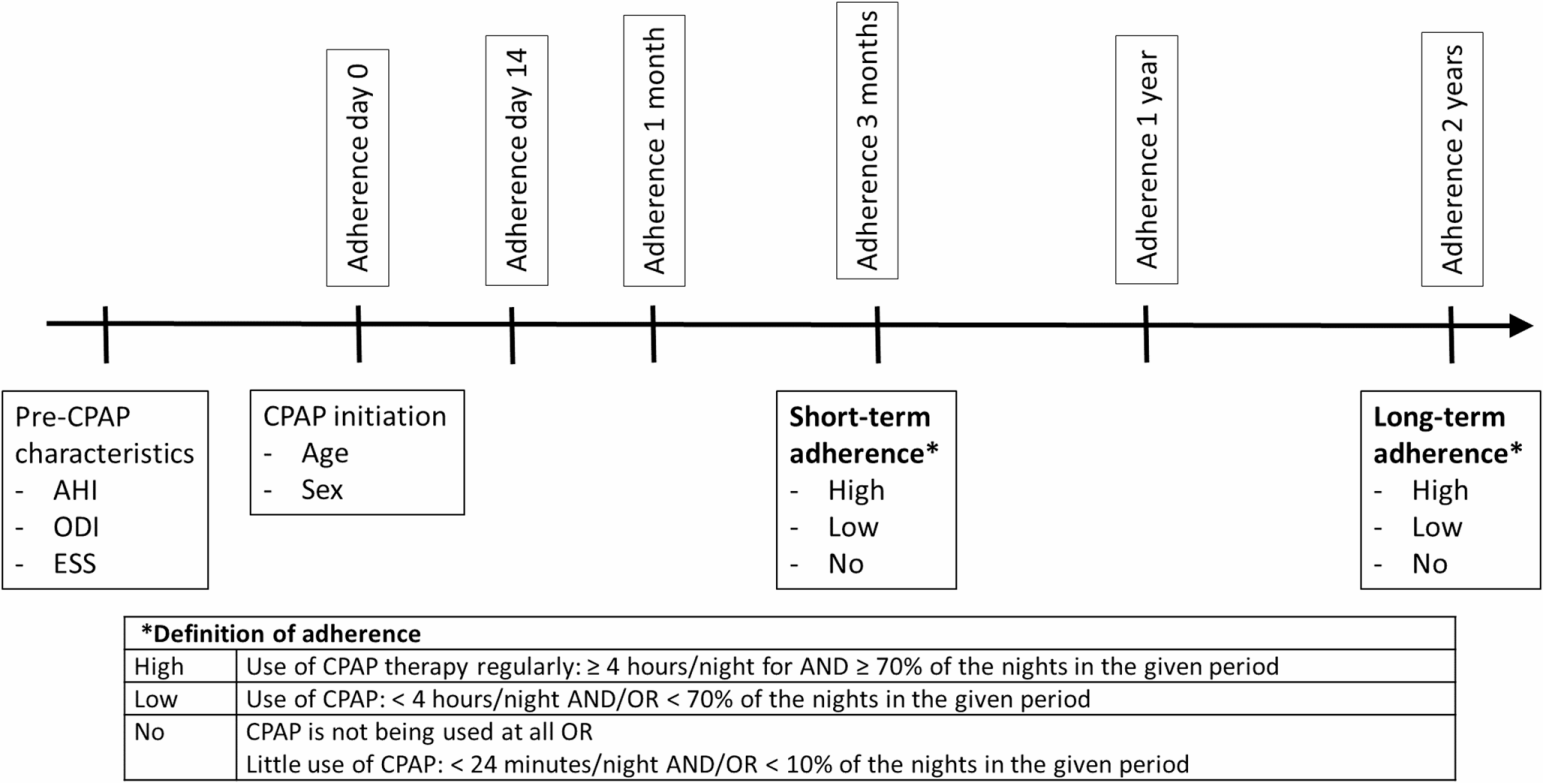
Timeline for CPAP Adherence Measurements. CPAP: continuous positive airway pressure, AHI: Apnea-Hypopnea Index, ODI: Oxygen Desaturation Index, ESS: Epworth Sleepiness Scale

The primary outcome variable was CPAP adherence. Patients were grouped into three levels of adherence: high, low, and no (Fig. 1). The definition of high adherence was based on the Centers for Medicare and Medicaid Services (CMS) criterion, which is CPAP use ≥ 4 h/night on ≥ 70% of nights over a given time period [18]. Using a clinical approach, the authors established that CPAP therapy usage below 10% of nights over the same time period would be classified as no adherence, distinguishing it from low adherence.

Missing adherence data were interpreted as nights where CPAP was not used and were categorised as zero-hour use. The long-term adherence classification was based on the adherence status at two years after CPAP initiation. Adherence was measured at 14 days, one month, three months, one year, and two years after CPAP initiation. Adherence was calculated using all available data (max 90 days) prior to measurement.

The following data were collected from DNPR:

- Age and sex at CPAP initiation.
- Pre-CPAP Body Mass Index [19].
- Pre-CPAP Apnea-Hypopnea Index (AHI) [20].
- Pre-CPAP Oxygen Desaturation Index (ODI).
- Pre-CPAP Epworth Sleepiness Scale (ESS) [6, 7].

### Statistical methods

Descriptive methods were used to present the baseline characteristics of the study population, distributed by levels of short-term adherence. Depending on their distribution, numerical variables are shown as mean (standard deviation, SD), and categorical covariates are presented as absolute and relative frequencies.

A Sankey diagram was developed to graphically illustrate patients’ distribution and movement patterns between adherence levels over time.

We used a multinomial logistic model to calculate the transition probabilities for changing adherence levels (high, low, and no adherence) between short-term and long-term adherence. We performed a sensitivity analysis of transition probabilities for changing adherence levels after 14 days, one month, three months, and one year of therapy to long-term adherence.

“Unlikely adherence data”, such as CPAP usage exceeding 24 h/day, were managed by replacing these values with the average of the likely available observations for the particular patient. In the case of missing values for AHI, ODI, and ESS, we assumed these values to be missing at random due to the nature of the data and study design and, therefore, did not replace them.

Statistical analyses were performed using Stata (College Station, Texas, USA: StataCorp LLC).

### Ethical considerations

The disclosure of patient record information without informed consent was based on a regional council approval according to Sect. 46, part 2 of the Health Act (22/60970). Approval for collecting data from the medical records was obtained from the management of involved hospitals, and the processing of personal data was notified and approved by the Region of Southern Denmark (23/4511) cf. art 30 of The EU General Data Protection Regulation. The study did not require Ethics Committee approval or informed consent under Danish law for register-based studies.

## Results

**Fig. 2:**
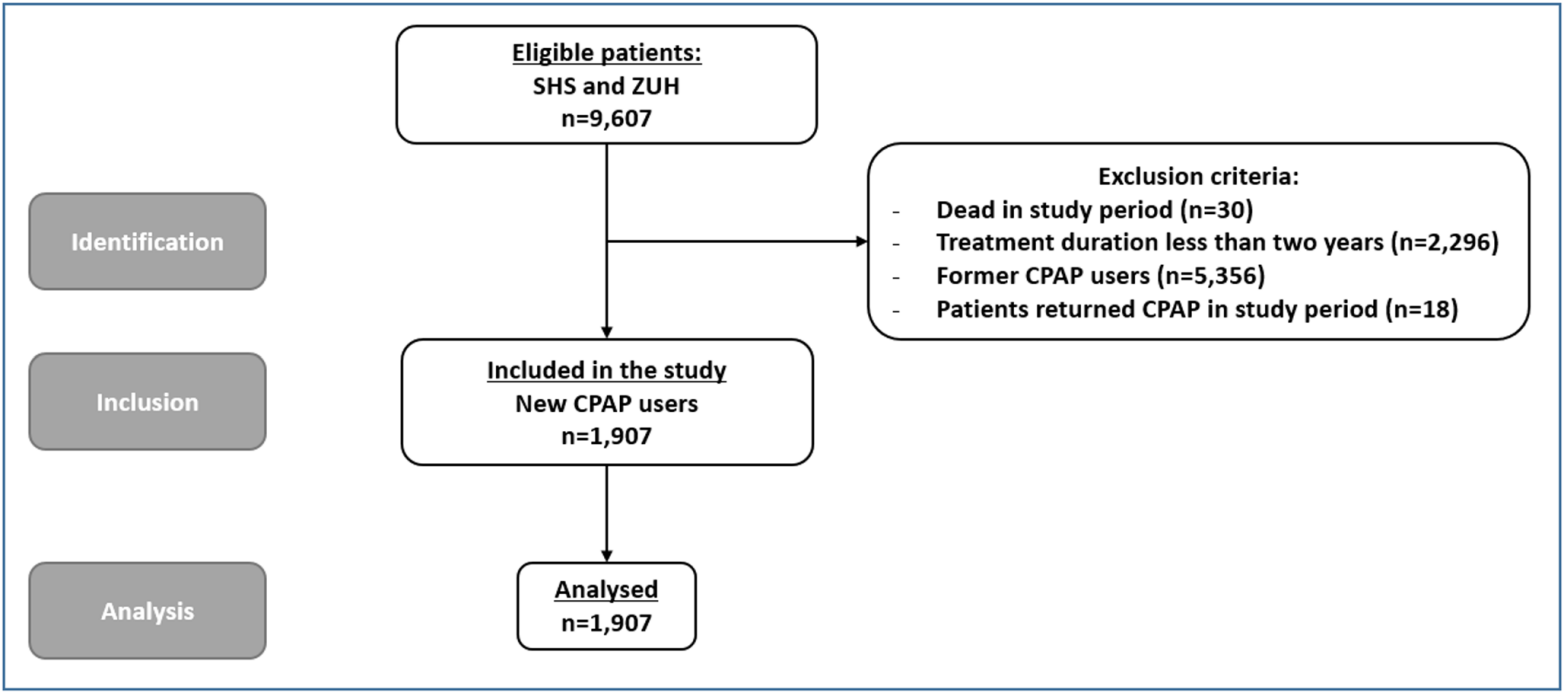
Study Enrolment Flow Chart. SHS: Sleep Clinic Hospital Sønderjylland in Sønderborg, ZUH: Sleep Clinic at the Zealand University Hospital in Køge

Of the 9,607 eligible patients, 1,907 were included in the study analysis (Fig. 2). During and after the COVID-19 period, many patients already receiving CPAP were offered a free replacement with a telemonitored device, allowing remote adjustments and follow-up without hospital visits. As a result, 5,356 (56%) of eligible patients were excluded due to this former CPAP user exclusion criterion.

Based on the AHI, most patients (95% of those treated with CPAP therapy) had moderate or severe OSA. In the Normal and mild categories for AHI/ODI, the majority of the patients were in the mild group, and only very few had a normal pre-CPAP AHI or ODI. We do not know why patients with a normal AHI/ODI were treated with CPAP. Since BMI was available for only 14% of the population, we decided not to use this variable in the analyses. Of the adherence data, 0.02% were categorised as “unlikely data” as described above in the method section.

**Table 1:** Patient characteristics at the time of therapy initiation distributed by levels of Short-term adherence

		High adherence^a^	Low adherence^a^	No adherence^a^	Total
		*n* = 740 (39%)	*n* = 502 (26%)	*n* = 665 (35%)	*n* = 1,907
Sex	Male	525 (70.9%)	369 (73.5%)	517 (77.7%)	1,411 (74.0%)
	Female	215 (29.1%)	133 (26.5%)	148 (22.3%)	496 (26.0%)
Age^b^		56.10 (11.93)	53.82 (11.96)	54.53 (13.37)	54.95 (12.49)
AHI pre-CPAP	Normal and Mild 0-14^c^	28 (4.5%)	15 (3.4%)	41 (6.9%)	84 (5.1%)
	Moderate 15–29	163 (26.2%)	120 (27.1%)	162 (27.5%)	445 (26.9%)
	Severe ≥ 30	432 (69.3%)	308 (69.5%)	387 (65.6%)	1,127 (68.1%)
ODI pre-CPAP	Normal and Mild 0-14^c^	40 (6.4%)	38 (8.5%)	44 (7.4%)	122 (7.3%)
	Moderate 15–29	179 (28.7%)	133 (29.9%)	181 (30.6%)	493 (29.7%)
	Severe ≥ 30	405 (64.9%)	274 (61.6%)	367 (62.0%)	1,046 (63.0%)
ESS pre-CPAP	Normal 0–7	145 (24.2%)	124 (29.0%)	152 (27.5%)	421 (26.7%)
	Mild 8–9	59 (9.9%)	37 (8.7%)	68 (12.3%)	164 (10.4%)
	Moderate 10–15	251 (42.0%)	163 (38.2%)	175 (31.7%)	589 (37.3%)
	Severe 16–24	143 (23.9%)	103 (24.1%)	157 (28.4%)	403 (25.6%)

a) Measured three months after CPAP therapy initiation

b) At CPAP therapy initiation, presented as mean (SD)

c) According to the General Data Protection Regulation (GDPR) in Denmark, patient counts below five must not be visualised in tables or figures in register-based studies; therefore, the groups of normal and mild pre-CPAP AHI and ODI were merged

CPAP: Continuous Positive Airway Pressure, AHI: Apnea-Hypopnea Index, ODI: Oxygen Desaturation Index, ESS: Epworth Sleepiness Scale

By subanalysis, we found that the majority of the patients (40–44%) from age 18 to 39 were non-adherent to CPAP, whereas patients from the age of 40 to 79 had a different pattern, with the majority of the patients achieving high adherence to CPAP (38–44%) (Appendix 1).

Twenty patients (1% of the cohort) underwent OSA-related surgery during the study period. Of these, 19 had a septoplasty and five had either a tonsillectomy or uvulopalatopharyngoplasty (some had a combination of procedures). Two years after CPAP initiation, 40% of these 20 patients had high adherence, 30% low and 30% no adherence to CPAP.

**Fig. 3:**
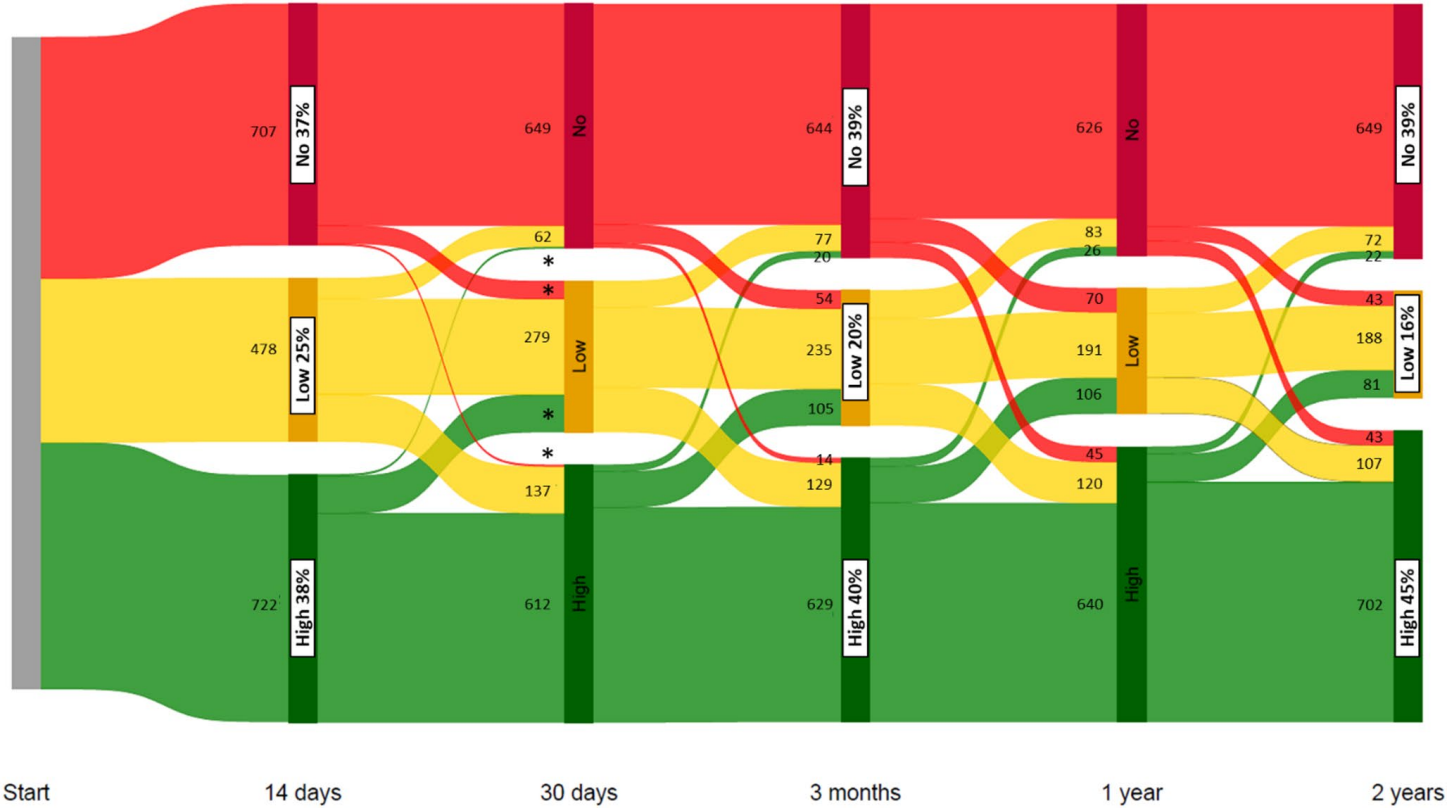
Sankey diagram. * According to the General Data Protection Regulation in Denmark, patient counts below five must not be visualised in figures in register-based studies. Therefore, data points have been omitted from this figure to ensure that it is impossible to calculate the hidden groups

Figure 3 shows the patterns of adherence groups and movements between these groups from the initiation of CPAP therapy and two years forward. The proportion of patients who are highly adherent to CPAP rises slightly during the two years of follow-up. The proportion of patients with no adherence is stable. This figure shows that some patients change the adherence grouping for each time interval.

**Fig. 4:**
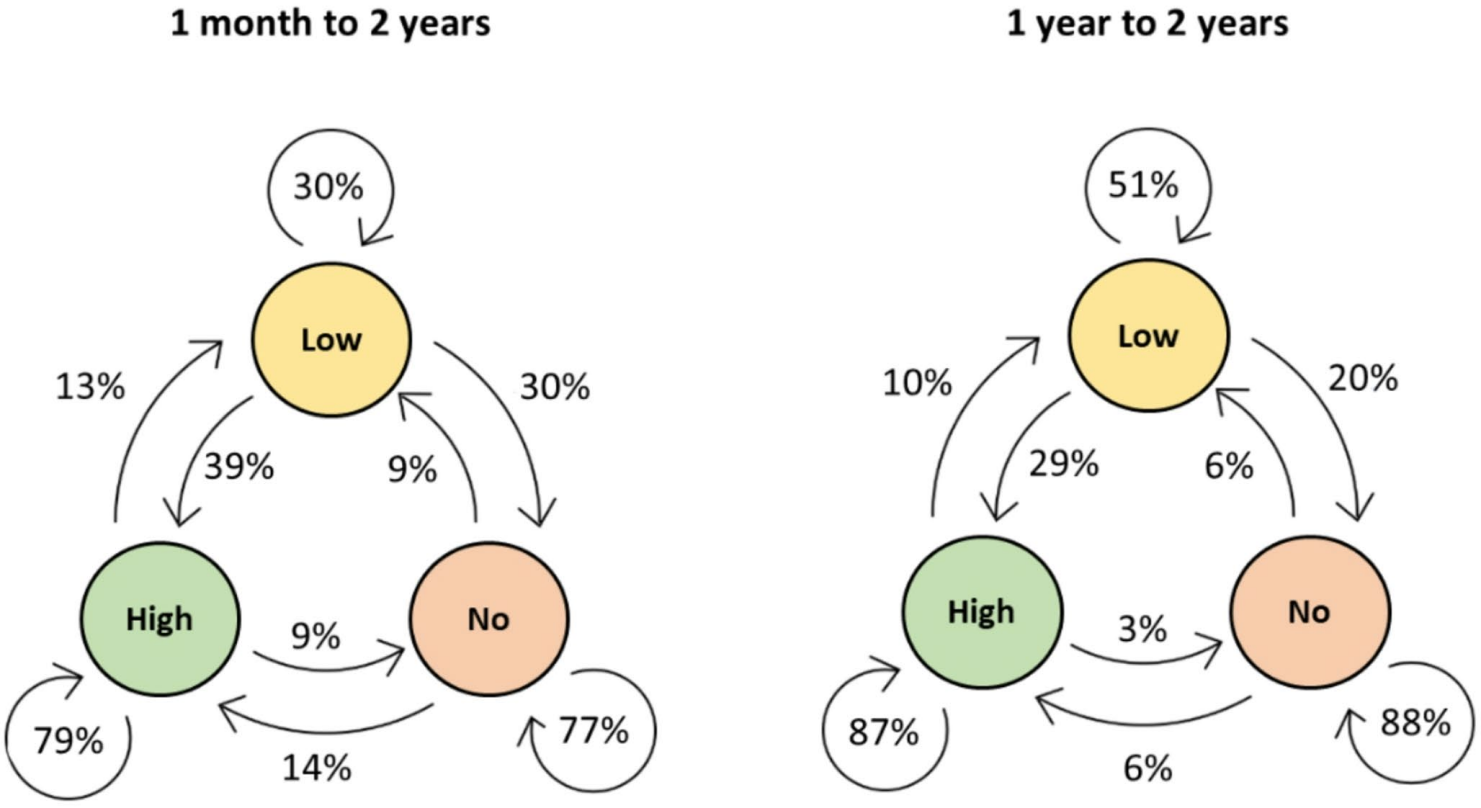
Transition probabilities

Figure 4 shows the probabilities for changing the adherence group from one month or one year to two years. The probabilities indicate that if a patient had high adherence after one month, the probability of having high adherence after two years was 79%. After one year, this probability increased to 87%. A similar increase was observed for the other transition probabilities for staying in the same group while changing groups decreased in probabilities.

We also calculated the transition probabilities for 14 days and three months to two years, which had similar results (Appendix 2).

**Fig. 5:**
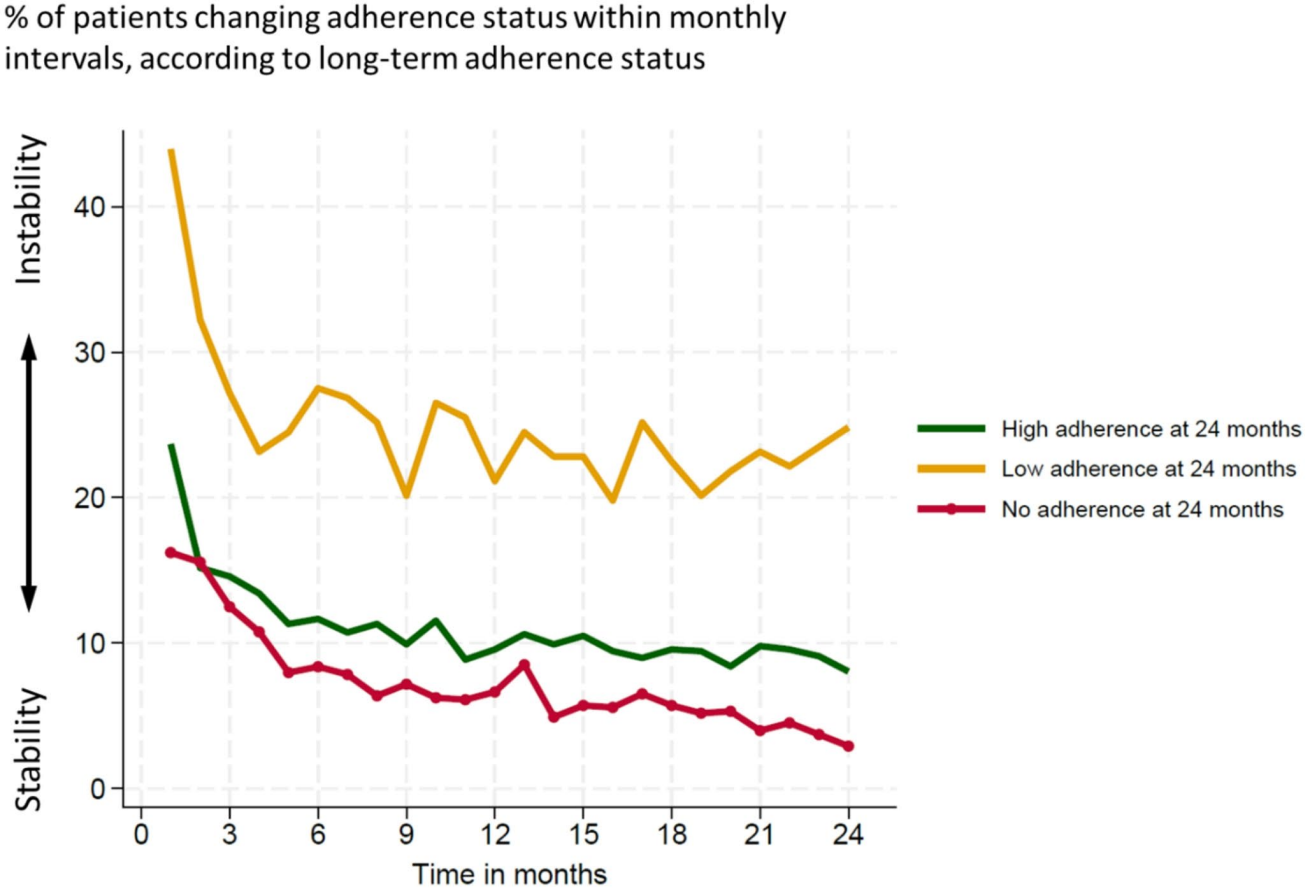
Stability of adherence groups

Figure 5 indicates that OSA patients are more unstable at the beginning of their treatment than later. For all three groups, the first point shows the percentage of the patients who changed adherence group between 14 days to one month, and then for each month until two years.

For the high adherence group, 23% changed from another adherence group (low or no adherence) between the first 14 days and one month. After two months, the high adherence group remained stable, with 10% changing groups throughout the next two years. The non-adherent group was more stable from the start, with an average of 7% changes. The low-adherence group was the most unstable from the beginning. It remained unstable throughout the two years of follow-up, with an average of 25% of the patients changing the adherence group each month.

## Discussion

From the patterns of longtime adherence to CPAP, we found that 45% of the patients were highly adherent two years after CPAP therapy initiation, 39% were non-adherent, and 16% had low adherence. The transition probability for remaining highly adherent between one month and two years was 79%, and remaining non-adherent was 77%. Stability of adherence levels occurred within the first three months for all patients. The highly and non-adherent groups were the most stable, while the low-adherent group remained unstable.

### Adherence patterns

When comparing adherence rates between studies, two elements must be considered: (1) the definition of high adherence and (2) excluding non-adherent patients. In this present study, we used the CMS criterion of ≥ 4 h/night of CPAP use for ≥ 70% of the observation period [18], which is an international and well-known definition and often clinically used to define when the patients are well treated (especially OSA patients who are professional drivers with a legal treatment requirement necessary for retainment of their driver’s licence). Using the same definition for high adherence, Cowen et al. [13] found a high adherence rate of 46% after 90 days and throughout the study period. Hamada et al. [21] reported a high adherence rate of 46.8% and 45.6% for 3 months after CPAP therapy initiation and 12 months, respectively. Yi et al. [22] found that after 3 months, the high adherence rate was 53.8%. All of these are very close to our findings in this study.

In contrast, Rotenberg et al. [7] reported their result from a systematic review on CPAP adherence as an overall CPAP non-adherence rate of 34.1% based on a 7-h/night sleep time, which makes comparison more difficult. Two large data analyses by Cistulli et al. [14] and Drager et al. [12] presented three-month high adherence rates of 75% and 66.4–74.0%, respectively. However, both studies excluded patients with no adherence to CPAP from the start; therefore, the adherence rates are not comparable. Further, Drager et al. only included patients who used the function MyAir^®^, thereby inducing selection bias in a more technically motivated cohort. Pépin et al. [23] showed decreasing adherence rates over three years using a national health insurance reimbursement system database and not adherence data. This decrease is not in line with our results, suggesting that our population has a more stable pattern of CPAP use.

Our study is pragmatic and, therefore, used data from all patients who had CPAP with prescribed telemonitoring. The group of non-adherent patients is clinically important and relatively large. This may partly reflect the Danish context, where CPAP is offered as the first-line treatment, and alternatives are typically considered after CPAP failure. As a result, some patients may initiate CPAP therapy without a strong motivation. In addition, adherence might have been higher if telemonitoring had been used proactively, like recent studies have shown improved CPAP use with this approach [24]. Future research should investigate if phenotyping non-adherent patients from the time of diagnosis might change the treatment choice with earlier consideration of other treatments such as MAD, positional trainers, surgery or a combination of these [25] or if they would benefit from a proactive telemonitored approach.

### Transition probabilities

In a Sankey diagram, it is impossible to see if the patients changing adherence groups over time are the same for each interval. Therefore, we calculated the transition probabilities at different time slots. We found that the probabilities for high adherence after two years were high if the patients were in the high adherence group at one month or one year, which is similar to the large study by Drager et al. [12], showing that > 80% with high adherence after three months were still using CPAP after one year. For the group with no adherence to CPAP, we found a similar result with a high probability of having no adherence to CPAP after two years if the patient had no adherence three months after CPAP initiation, which indicates stabilisation at some point.

### Time for stabilisation

Several studies have, in line with our results, reported early establishment of adherence levels after initiation of CPAP therapy [22, 26–28]. Our study showed no further adherence improvement between one and two years. Even though the high and non-adherent groups stabilised, around 90% and 93%, respectively, up to 10% were still unstable at any given time up until two years. These patients may indicate a sub-group of patients close to the adherence cut-offs. The group with low adherence became more stable over the two years of follow-up. This is a particularly relevant group to consider stabilising as we want them to become highly adherent users or to try another treatment option. Compared to the results in the Sankey Diagram, the group of low-adherent patients decreased in the follow-up time. It is relevant to identify these patients early after initiating CPAP therapy since they may need professional help to achieve high adherence. The low adherent classification describes a patient group trying to use the CPAP therapy but struggling to reach a goal of everyday use or enough hours/night. With close monitoring and help within 14 days after therapy initiation, where the low adherence pattern begins, we may be able to help more patients become high adherence CPAP users.

### The definitions of adherence groups

Several studies have questioned whether the CMS criteria are the optimal dose-dependent limit for high adherence in CPAP-treated OSA patients [7, 29]. It is challenging to determine precisely when the CPAP therapy is sufficient to reduce tiredness, give full awareness in traffic, and affect comorbidities. Studies have shown that even CPAP use for lower than four hours/night was associated with better outcomes, such as reduced self-reported tiredness and lower healthcare utilization, compared to no CPAP use. These findings suggest that the current 4 h/night limit might need to be taken up for re-evaluation [29, 30]. With the increasing availability of telemonitored CPAP using data, future research may identify more personalised adherence limits, potentially leading to tailored alternatives to the current CMS criteria.

Further, the limit for no use versus low adherence is also a subject for discussion, though it has received little attention in the international literature. The relevance of this lower limit is questionable since patients who achieve no adherence or very low adherence to CPAP should naturally be offered another treatment or additional support. However, we found that the chances of achieving high adherence if a patient was in the non-adherent group after one year was only 6%. On the contrary, we found that 29% of the patients from the low-adherence group changed to the high-adherence group.

### Limitations

This study only represented patients who accepted CPAP devices with telemonitoring technology during the study period. Patients who chose a CPAP without telemonitoring could be elderly people, have reduced digital literacy, limited internet access, feel uncomfortable with being monitored, or lack trust in remote data collection. These factors represent a selection bias that may limit the generalisability of our findings. Further, the definition of no data from the CPAP machines as a measure for no use could be wrong, e.g., if the patient had put the CPAP machine on flight mode. However, we would expect that the proportion of patients with CPAP devices equipped with telemonitoring who chose not to transmit data was limited. Patients who by themselves bought a MAD, or experienced weight loss and subsequently no longer required CPAP therapy but did not return their CPAP machines to the sleep clinics were not distinguished in this analysis. These patients could potentially be included in the non-adherent group. Finally, BMI data were only available for 14% of patients, which prevented meaningful analysis. This is a limitation, as BMI may be an important predictor of CPAP adherence.

### Strengths

This study was a multicenter study that included long-term everyday adherence data on 1,907 patients. The population is representative of the general OSA population in Denmark and possibly other countries. The adherence data were combined with information from the patients’ medical records, which enabled a detailed investigation of the key factors influencing CPAP treatment usage, including frequency, timing, and patient characteristics. Unlike other studies investigating adherence data from telemonitored CPAP therapy [12, 14], we included data from patients who were non-adherent to CPAP from the initiation of the therapy, representing the patients visiting the clinics.

### Perspective and future research

Future studies should investigate the minimum dose-dependent limit for the benefit of CPAP. An increasing number of patients are choosing telemonitored CPAP therapy, which allows access to large datasets. Further phenotyping and prediction studies focusing on comorbidity and socioeconomic factors in OSA patients are needed to better predict adherence patterns early in the treatment process. This could help clinicians to present a more individualised treatment, as patients predicted to have high adherence may not require the same treatment protocol or follow-up as those expected to have low or no adherence. Like in other recent research [31], gender differences could also be interesting to explore in future research on patterns and stability in CPAP adherence.

To better capture the dynamics of CPAP adherence over time, trajectory modelling would be beneficial. This could offer a more nuanced and statistically robust framework for understanding latent adherence patterns.

## Conclusion

The long-term adherence patterns to CPAP therapy revealed that 45% of patients were highly adherent two years after initiation. Patients who achieved high adherence within the first month were likely to maintain it long-term, while non-adherent patients were likely to remain so. This stability in adherence levels was established within the first few months after CPAP initiation. We confirm the potential of early usage data to support treatment adjustments and ensure long-term high adherence, as well as to identify patients needing additional assistance or alternative treatment options.

## Electronic supplementary material

Below is the link to the electronic supplementary material.

**Supplementary Material 1**: **Appendix 1**: age at cpap therapy initiation by Levels of Short-term Adherence.

**Supplementary Material 2**: **Appendix 2**: Transition probabilities for changing the adherence group from 14 days or three months to two years.

## Data Availability

The datasets generated and analysed during the current study are not publicly available in accordance with Danish law and due to personal information. Data are available from the corresponding author on reasonable request.
