# Supplementary material for: Patterns and stability of long-term adherence in continuous positive airway pressure therapy for obstructive sleep apnea: a cohort study
Source: Sleep Breath. 2025 Jul 15;29(4):243. doi: 10.1007/s11325-025-03418-9 (PMC12263751; doi:10.1007/s11325-025-03418-9)
Supplement: Supplementary file 1 — Supplementary Material 1: Appendix 1: age at cpap therapy initiation by Levels of Short-term Adherence. [file 11325_2025_3418_MOESM1_ESM.docx]

appendix 1: age at cpap therapy initiation by Levels of Short-term Adherence

| **Age at CPAP therapy start** | **High adherence*** | **Low adherence*** | **Low adherence*** | **Total** |
| --- | --- | --- | --- | --- |
| 18-29 years | 18 (31.58%) | 16 (28.07%) | 23 (40.35%) | **57 (100%)** |
|  | 2.43% | 3.19% | 3.46% | **2.99%** |
| 30-39 years | 46 (26.44%) | 52 (29.89%) | 76 (43.68%) | **174 (100%)** |
|  | 6.22% | 10.36% | 11.43% | **9.12%** |
| 40-49 years | 145 (38.98%) | 101 (27.15%) | 126 (33.87%) | **372 (100%)** |
|  | 19.59% | 20.12% | 18.95% | **19.51%** |
| 50-59 years | 228 (37.75%) | 178 (29.47%) | 198 (32.78%) | **604 (100%)** |
|  | 30.81% | 35.46% | 29.77% | **31.67%** |
| 60-69 years | 199 (43.64%) | 109 (23.90%) | 148 (32.46%) | **456 (100%)** |
|  | 26.89% | 21.71% | 22.26% | **23.91%** |
| 70-79 years | 92 (43.19%) | 40 (18.78%) | 81 (38.03%) | **213 (100%)** |
|  | 12.43% | 7.97% | 12.18% | **11.17%** |
| 80 years or older | 12 (38.71%) | 6 (19.35%) | 13 (41.94%) | **31 (100%)** |
|  | 1.62% | 1.20% | 1.95% | **1.63%** |
| **Total** | **740 (38.80%)** | **502 (26.32%)** | **665 (34.87%)** | **1,907 (100%)** |

* Measured three months after CPAP therapy initiation

Data are presented as n (%)
