# Supplementary material for: Patterns and stability of long-term adherence in continuous positive airway pressure therapy for obstructive sleep apnea: a cohort study
Source: Sleep Breath. 2025 Jul 15;29(4):243. doi: 10.1007/s11325-025-03418-9 (PMC12263751; doi:10.1007/s11325-025-03418-9)

### appendix 2: Transition probabilities for changing the adherence group from 14 days or three months to two years


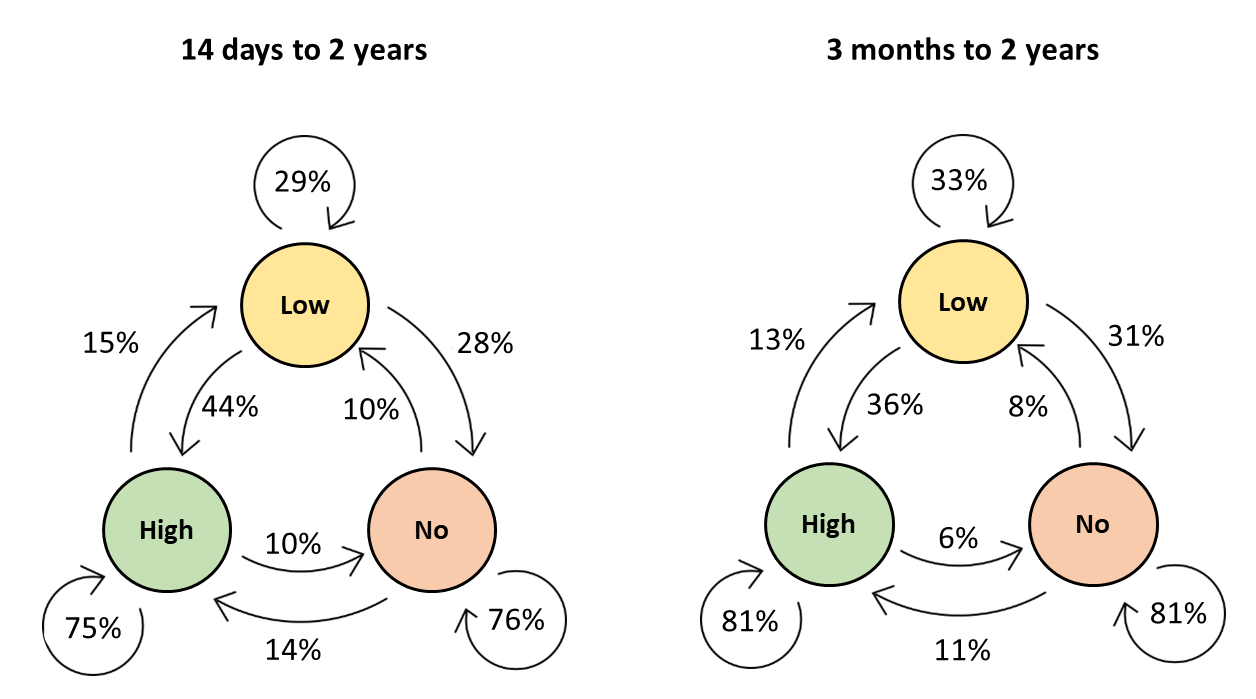

Supplement: Supplementary file 2 — Supplementary Material 2: Appendix 2: Transition probabilities for changing the adherence group from 14 days or three months to two years. [file 11325_2025_3418_MOESM2_ESM.docx]
